# Analysis of Conformational Preferences in Caffeine

**DOI:** 10.3390/molecules27061937

**Published:** 2022-03-17

**Authors:** Sara Gómez, Natalia Rojas-Valencia, Albeiro Restrepo

**Affiliations:** 1 Classe di Scienze, Scuola Normale Superiore, Piazza dei Cavalieri 7, 56126 Pisa, Italy; 2 Instituto de Química, Universidad de Antioquia (UdeA), Calle 70 No. 52-21, Medellin 050010, Colombia; nandrea.rojas@udea.edu.co

**Keywords:** caffeine, NBO, hyperconjugation, methyl rotation

## Abstract

High level DLPNO–CCSD(T) electronic structure calculations with extended basis sets over B3LYP–D3 optimized geometries indicate that the three methyl groups in caffeine overcome steric hindrance to adopt uncommon conformations, each one placing a C–H bond on the same plane of the aromatic system, leading to the C–H bonds eclipsing one carbonyl group, one heavily delocalized C–N bond constituent of the fused double ring aromatic system, and one C–H bond from the imidazole ring. Deletion of indiscriminate and selective non-Lewis orbitals unequivocally show that hyperconjugation in the form of a bidirectional –CH${}_{3}\;⇆$ aromatic system charge transfer is responsible for these puzzling conformations. The structural preferences in caffeine are exclusively determined by orbital interactions, ruling out electrostatics, induction, bond critical points, and density redistribution because the steric effect, the allylic effect, the Quantum Theory of Atoms in Molecules (QTAIM), and the non-covalent interactions (NCI), all predict wrong energetic orderings. Tiny rotational barriers, not exceeding 1.3 kcal/mol suggest that at room conditions, each methyl group either acts as a free rotor or adopts fluxional behavior, thus preventing accurate determination of their conformations. In this context, our results supersede current experimental ambiguity in the assignation of methyl conformation in caffeine and, more generally, in methylated xanthines and their derivatives.

## 1. Introduction

Caffeine is a psychoactive drug of the xanthines family with proven health and cognition benefits. Caffeine is the basis of a multibillion industry world wide and belongs to a short list (nicotine, theobromine, theophylline, etc.) of alkaloids that are legal to consume without limitations but whose content in food, beverages, and supplements is regulated by the FDA [1]. Synthetic routes are available [2] to produce the industrial quantities needed for the global demand that caffeine of natural origins cannot satisfy. The scientific literature covering all aspects of caffeine is vast [3,4,5,6,7,8].

**Figure 1:**
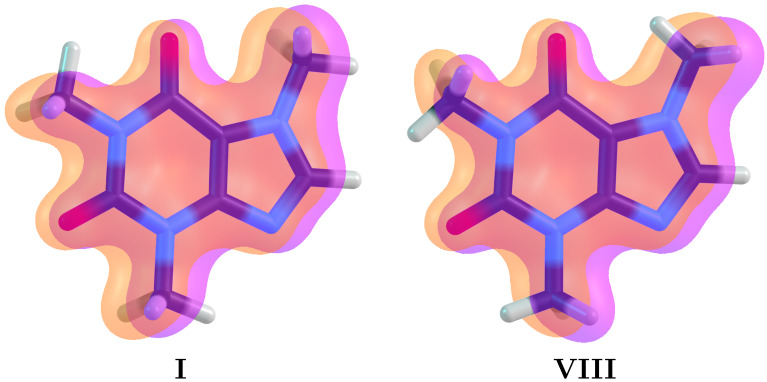
Extended π delocalization for the lowest (**I**) and highest (**VIII**) energy structures in caffeine. This delocalization contains a ten π−electron aromatic core from the two fused rings and contributions from the carbonyl groups. Notation: M1 is the methyl group at the top left of the pyrimidine ring, M2 is the methyl group at the bottom of the pyrimidine ring and M3 is the methyl group at the imidazole ring.

Molecular structure determines molecular function and most of the properties of substances, including metabolism and biological action, therefore, accurate determination of molecular structure is certainly one of the top priorities of chemists working in every sub field. All heavy atoms in caffeine molecules are coplanar yielding a ten π–electron aromatic core [9,10,11], however, the positions of the hydrogen atoms in the three methyl groups have not been unambiguously determined. A number of factors contribute to the solid and liquid structure of caffeine, among them π-π stacking seems to play a pivotal role. In this regard, studies of the structures of dimers and trimers indicate that the methyl groups from the interacting molecules are placed as far apart from each other so as to maximize the interaction between the two π clouds [5]. Additional studies explore the role of up to five explicit water molecules in the structure of the dimers and found that at those low molecularities the water molecules prefer the polar regions of caffeine [12]. Figure 1 shows the associated π cloud which will be discussed later and serves to introduce a useful notation: in what follows, we refer to the methyl groups as M1 (top left at the pyrimidine ring), M2 (bottom of the pyrimide ring), and M3 (at the imidazole ring). Notice that all methyl groups have different chemical environments and thus each internal rotation must be analyzed in detail. Within caffeine, methyl rotations are heavily influenced among other factors by the 1,3–allylic effect [13,14] (M3 is separated by four atoms from the nearest carbonyl group and thus is only affected by remote allylic interactions), by the π density of the heavily conjugated system, by the ability to form intramolecular CH⋯π interactions [15], and by the well known ability of C–H bonds in methyl groups to eclipse C–H bonds in aromatic planes [16,17].

The ambiguity in the experimental position of the hydrogen atoms arises from two sources. First, in the crystallographic reports [18,19,20], the methyl groups are held in frozen positions within the solid state structures. Second, in the gas phase neutron scattering [21] and electron diffraction experiments [22], the positions of the methylic hydrogens are derived after fitting the internal rotations to empirically constructed rotational energy profiles. Microwave spectroscopy would certainly remove the experimental uncertainty in the location of the hydrogen atoms in methyl groups in caffeine, however, such studies are not available. In this scenario, honoring Frank Weinhold on occasion of his 80th birthday, we resort to NBO and other analysis methods to resolve this structural conundrum. NBO, developed over the last few decades in the group of Frank Weinhold [23,24,25,26,27,28,29,30,31,32,33], is a widely used and highly successful strategy in the analysis of a large body of chemical problems [34,35,36,37,38,39,40,41,42,43,44,45,46,47,48,49,50,51,52,53,54,55,56,57,58,59,60]. Specifically, for the conformational problem in caffeine, for each methyl rotation, we study hyperconjugation, electron delocalization involving the methyl groups, NBO donor–acceptor interactions, and the effect of all these factors on geometrical variables. In this manuscript, not only do we help removing the present ambiguity in the determination of the conformation of the methyl groups in caffeine, but we also clearly identify the causes for these structural preferences.

## 2. Methods

In order to have an unbiased picture of the structural preferences for methylic hydrogens in caffeine, we ran relax scans in the following way: M3 is far removed from the other two methyl groups (Figure 1), thus, this rotation was explored individually, however, the two methyl groups in the pyrimidine ring are connected via the intermediate carbonyl group, therefore, we computed two dimensional relaxed rotations for M1, M2 for both possible eclipsed conformations of the C–H bond at M3 (eclipsing a carbonyl or eclipsing a ring C–H) at the B3LYP–D3/6–311++G(d,p) level, chosen because it has proven accurate and efficient in structural and spectroscopic studies of a family of related xanthines [61,62]. All stationary points afforded by these scans were then optimized while freezing the particular conformation of the methylic C–H bonds and characterized as either true minima or saddle points at the same level. Determining explicit interactions of methyl groups with environmental molecules would require detailed explorations of large potential energy surfaces [47], as a first approximation to this issue, we study, under continuum models, structural and energetic effects of solvation with water, acetone, acetonitrile, chloroform, dichloromethane and toluene, a series of solvents for which explicit experimental solubilities are available [63,64]. Highly correlated DLPNO–CCSD(T) energies with a tight convergence criterion [65] were also calculated on the stationary points to establish accurate relative energies. CCSD(T) is the present golden standard for accuracy in computational chemistry as it recovers > 99.7% of electron correlation (See Table 11.7 in Ref. [66]), unfortunately, CCSD(T) is extremely expensive as it scales with the size of the system as $N^{7}$ (see Table 7.5 in Ref. [67]), thus, CCSD(T) calculations with extended basis sets are prohibitive even for moderate size molecules. DLPNO–CCSD(T) is an efficient alternative scheme that recovers >99.9% of the pure CCSD(T) energies at the cost of a typical DFT calculation and scales linearly with *N* [68,69]. Reassuringly, Sandler and coworkers [70] very recently explicitly stated “*The errors in the DLPNO–CCSD(T) were found to be relatively insensitive to the choice of basis set for small systems but increase monotonically with system size*”. All scans, geometry, frequency and solvent calculations were carried out using the Gaussian16 suite of programs [71]. DLPNO–CCSD(T) energies were computed using the ORCA 4.1.2. program [72].

**Figure 2:**
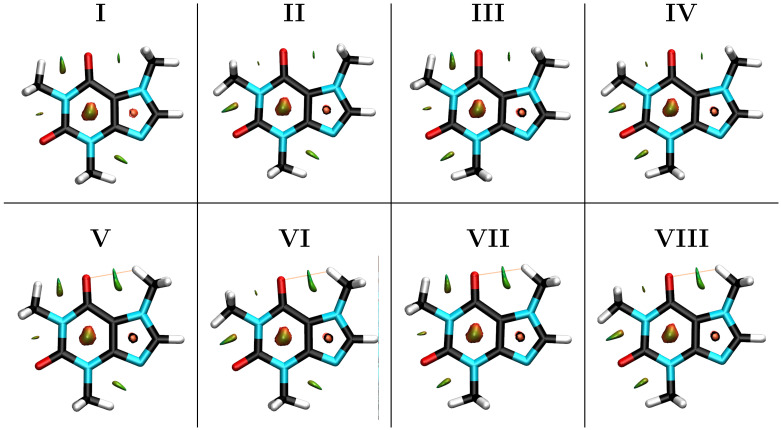
Eight possible structural conformers of caffeine holding one C–H bond from each methyl group in the aromatic plane, as determined from relaxed scans for the rotation of each methyl group. NCI green surfaces and bonding paths associated to the in–plane C–H bonds from the methyl groups are shown. NCI red surfaces showing the strong non–bonded overlap at the center of the aromatic cycles are also shown.

Once the structural problem is solved by the above methods, we analyze structural preferences using NBO [23,28] as implemented in NBO7 [73], QTAIM [74] as implemented in AIMAll [75] to determine if the in–plane hydrogen atoms from the methyl groups interact with other atoms, and NCI [76] as implemented in NCIPLOT [77] for the same purpose. Two formal NBO strategies are employed:

1. Hyperconjugation effects on electronic energies:
  (a) The energy of a localized Lewis structure was constructed by deleting all non–Lewis orbitals from the Fock matrix and then the hyperconjugative contributions are obtained as the difference between the full and localized structures. This procedure was repeated for each one of the eight conformers located in this work as shown in Figure 2 and for the relaxed scans involving interconversion of **I** via the two lowest energy paths afforded by the structural analysis, namely **I→II→I** and **I→III→I**.
  (b) Specific hyperconjugative contributions from the methyl groups to the eight conformers in Figure 2 were obtained by deletion of all donor→acceptor interactions involving the methyl groups.
2. Hyperconjugation effects on molecular geometries: following a strategy suggested elsewhere [29,78,79,80,81,82], geometry optimizations for the rotations of the methyl groups within the lowest energy structure were carried out by deleting all hyperconjugative interactions involving each methyl group separately and then involving all methyl groups simultaneously.

## 3. Results and Discussion

### 3.1. Validation of the Calculations

Besides the existing literature that supports the use of B3LYP–D3/6–311++G(d,p) and other DFT methods to study caffeine [4,62], our own calculations solidify this choice of model chemistry. First, bond distances and angles perfectly match experimental data obtained using gas electron diffraction [22] as illustrated with the following four examples: the central C–H bond at the imidazole ring was measured at 1.085 Å, our calculations afford 1.080 Å. The experimental values of the C–N bonds attaching M1, M2, and M3 to the fused rings are 1.464, 1.458 and 1.456 Å, the calculated values are 1.469, 1.464, and 1.459 Å, respectively. Second, the rotational barrier for M1 was estimated by Kim and coworkers [61] after fitting the experimental data to model potentials at ≈0.286 kcal/mol, our own B3LYP–D3 calculations afford 0.28 kcal/mol (see a detailed discussion of rotational barriers below).

### 3.2. Aromaticity

The aromatic character of the pyrimidine/imidazole fused rings in caffeine has been thoroughly discussed in the literature [9,10,11], with every single descriptor (NICS, ring current, FLU, electron delocalization) pointing to a highly delocalized ten π−electron system. Our own calculations show that the aromatic π core is further stabilized with contributions from the two peripheral oxygen atoms constituent of the double C=O bonds in localized Lewis structures. This extended delocalization of the π cloud, which is present in every single conformer and is illustrated for the lowest (**I**) and highest (**VIII**) energy structures in Figure 1, plays a crucial role in the hyperconjugative interactions that dictate the structural preferences for the methyl hydrogens, as discussed below.

### 3.3. Structures and Energies

**Figure 3:**
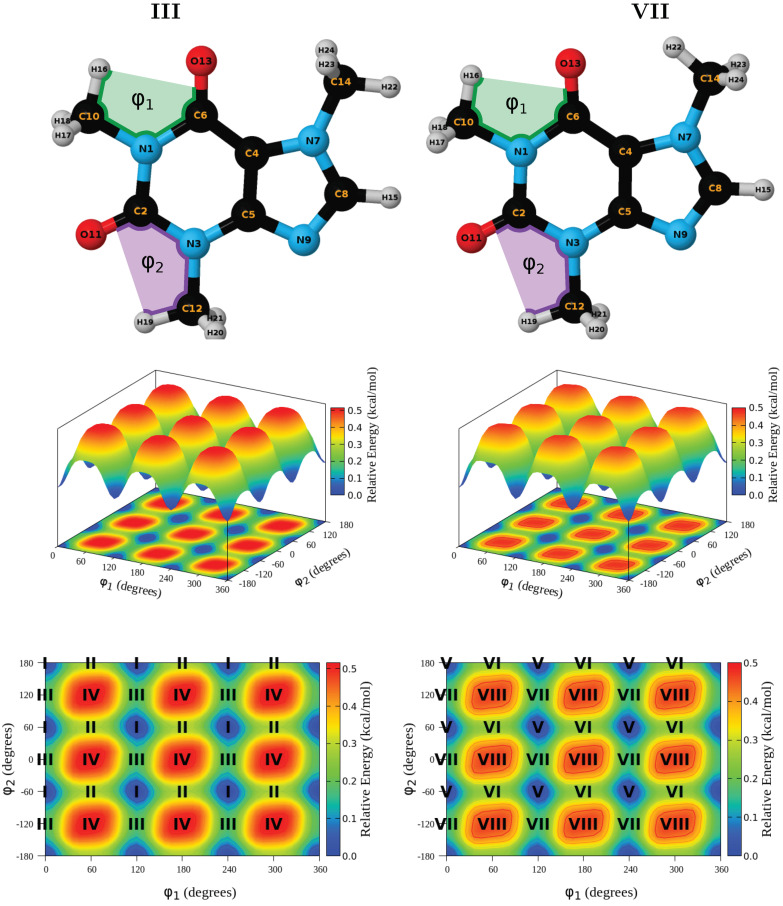
Two dimensional B3LYP–D3/6–311++G(d,p) relaxed scans to determine the conformations of the two methyl groups (M1, M2) attached to the pyrimidine ring in the two possible conformations of the methyl group attached to the imidazole ring (M3) in caffeine.

The one dimensional scans for the rotation of the methyl group at the imidazole ring afforded two groups of structures shown in Figure 2, with **I** and **V** having the lowest energy within each group. A pictorial summary of the bidimensional scans for the coupled rotations of the methyl groups at the pyrimidine ring is shown in Figure 3, the corresponding energies for all conformations, relative to **I**, the lowest energy structure at all levels of theory, are provided in Table 1. There are a few remarkable structural preferences whose study are the focus of this work.

Most intriguing is that, overcoming steric hindrance, structures with bond eclipsing, that is, with C–H bonds drawn into the aromatic plane, are energetically favored to the point that conformations in which all methylic hydrogens are placed off-plane do not even correspond to stationary points within the potential energy surface. A C–H bond in the methyl attached to the imidazole ring eclipses a C–H in the ring. This is highly unusual because if eclipsing of bonds occurs, C=O in pyrimidine to C–H in the imidazole methyl eclipsation (structures **V–VIII**) should be highly favorable due to a strong inductive effect, although the pyrimidinic carbonyl is four atoms apart so this is not exactly the 1,3-allylic effect [13,14]. This puzzling C–H/C–H in plane eclipsation has already been observed in thymine and was rationalized as being due to hyperconjugation in the form of charge transfer from the aromatic ring to the methyl group and vice versa [16]. There is also C–H/C=O eclipsing (1,3–allylic effect) in the pyrimidyne ring, however, only one of the methyl groups behaves this way (structures **III, IV, VII, VIII**), the second methyl prefers to eclipse a C–N bond in the imidazole ring (structures **I, II, V, VI**).

Small rotational barriers serve as a deep probe to understand at a fundamental level the inter and intra molecular forces behind structural preferences. From an experimental stance, even conformations separated by tiny rotational barriers as those in the present case can be unveiled by microwave spectroscopy. The point is that injection of a puff of a gaseous sample into a large vacuum cavity will lead to an expansion of the sample such that molecular collisions cease to occur, leading to cooling of individual molecules to rotational temperatures just a few degrees above 0 K, effectively freezing the molecules in the preferred conformation (for a detailed discussion please see the experimental section in Ref. [83]). According to Table 1, in the gas phase, all conformers are separated by very small energies. If purely electronic energies are considered, no conformer is separated by more than 0.9 kcal/mol from **I** at the DFT level or by 1.5 kcal/mol using CCSD(T), if vibrational energies are considered, those differences barely exceed 0.7 or 1.3 kcal/mol, however, when temperature, entropy and internal degrees of freedom are included, the difference increases to 3.1 kcal/mol under the DFT Gibbs free energies. The energetic picture is not altered in any sensitive way after the inclusion of the continuum solvent or after improving the basis set to aug–cc-pVTZ, whose largest DLPNO–CCSD(T) energy difference with respect to **I** in the gas phase is ≈1.8 kcal/mol. These numbers indicate that, given the accuracy of computational methods, and since $K_{B}T=0.6$ kcal/mol at room conditions, all methyl groups should either act as free rotors or exhibit fluxional behavior [39]. Indeed, internal rotational barriers for M1, M2, M3 have been reported on the order of 0.25, 0.15, 0.45 kcal/mol, respectively [62], furthermore, interconversion paths calculated in this work also afford very small barriers, the smallest one being 0.8 kcal/mol under Gibbs energies for **I→III→I**, similarly, 1.1 kcal/mol are obtained for **I→II→I**. In this context, small imaginary vibrational frequencies associated with methyl rotations are irrelevant. Another important point is that energy differences for the (**I**, **V**), (**II**, **VI**), (**III**, **VII**), (**IV**, **VIII**) pairs consistently afford ≈0.8 kcal/mol differences in purely electronic CCSD(T) energies, thus, it should be clear that the methyl rotation at the imidazole ring is unaffected by the distant conformations.

### 3.4. Hyperconjugation Effects on Electronic Energies

**Figure 4:**
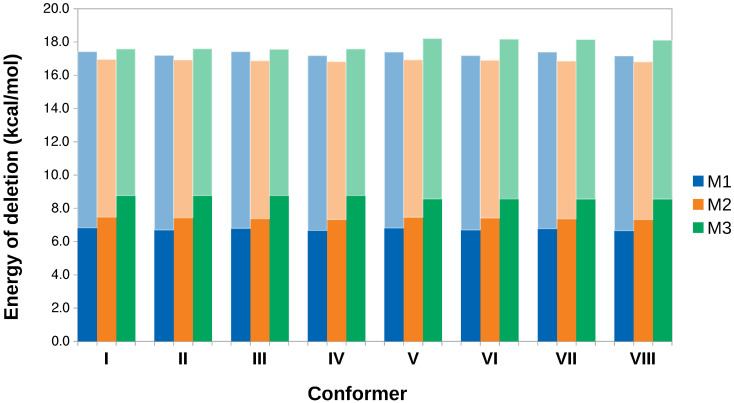
Hyperconjugation contributions to the conformations of the methyl groups in caffeine (M1, M2, M3 in Figure 2). Dark rectangles correspond to deletions of the NL orbitals involved only in charge donation from the methyl groups, light boxes account for the energy associated when deleting NL orbitals in the methyl groups when those groups act as charge acceptors.

**Figure 5:**
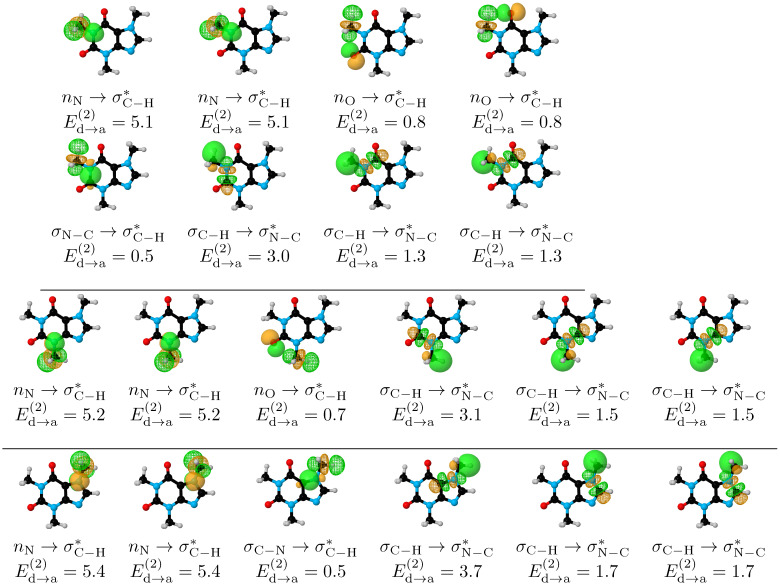
Donor (solid surfaces) → acceptor (meshed surfaces) orbital interactions for the conformer **I** of caffeine. All interactions are of the vicinal type (no geminal or remote hyperconjugation). All energies in kcal/mol.

We deleted all non-Lewis orbitals for each conformer and list the resulting energies in Table 2. Except for **III**, deletion of non-Lewis orbitals switches the energy values in detriment of **I**, clearly indicating that hyperconjugation is behind the structural preference of caffeine. Hyperconjugation accounts for charge transfer from Lewis to non-Lewis orbitals in the entire molecule, thus, to analyze the specific effect of the methyl groups, Figure 4 quantifies the energy associated with selective deletion of the orbitals affecting methyl conformation for each conformer. The combined length of the box quantifies the energy associated with deleting all non-Lewis (NL) orbitals involved in methyl donation and reception of charge, dark rectangles correspond to deletions of the NL orbitals involved only in charge donation from the methyl groups (deletions away from –CH${}_{3}$ as we did not find any geminal hyperconjugation), light boxes account for the energy associated when deleting NL orbitals in the methyl groups when those groups act as charge acceptors. The 20 orbital pairs involved in methyl donation and reception of charge are shown in Figure 5 for **I**.

For all methyl groups in caffeine, there is charge transfer in both directions (Figure 5), which may be schematized as –CH${}_{3}⇆$ double ring. This observation is rationalized by recalling that –CH${}_{3}$ is a strong σ electron donor group, thus, after donation, the non–Lewis $\sigma_{C-H}^{*}$ orbitals are activated to act as acceptors of charge. Based on this bidirectional charge transfer, NBO unambiguously explains why a C–H bond from the methyl group prefers to eclipse either a C=O group (M1, M2) or a C–H bond in the aromatic plane (M3), as follows. For the –CH${}_{3}\to$ double ring charge transfer, the largest contributors are always of the $\sigma_{C-H}\to \sigma_{N-C}^{*}$ form (notice that the acceptor $\sigma_{N-C}^{*}$ orbitals are not the same for the three methyl groups). Fittingly, $E_{d\to a}^{\left(2\right)}$ for the in-plane interactions in the three methyl groups (3.0, 3.1, 3.7 kcal/mol for M1, M2, M3, respectively) are so large that, for a particular methyl, this interaction actually exceeds the sum of all other –CH${}_{3}\to$ double ring combined. In addition, for the double ring → –CH${}_{3}$ charge transfer, the in-plane C–H bonds from the methyl groups position the three $\sigma_{C-H}^{*}$ orbitals such that two strong stabilization factors are also favored: the overlap between the donor $n_{N},n_{O}$ orbitals and the two out-of-plane acceptor $\sigma_{C-H}^{*}$ orbitals is maximized as seen in Figure 5, and the remaining in-plane $\sigma_{C-H}^{*}$ is perfectly located to now interact with three donor orbitals in M1, and with one donor orbital in the M2, M3 cases.

**Figure 6:**
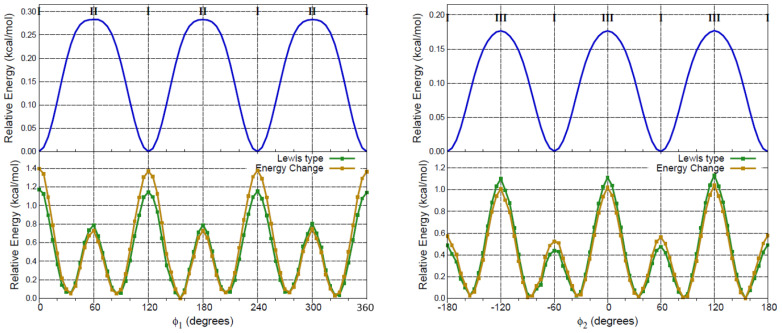
B3LYP–D3/6–311++G(d,p) energy profiles for the interconversion of **I** into itself via the two lowest energy barrier paths (see Figure 3). **Top**: full electronic energy paths. **Bottom**: deleting all hyperconjugative interactions (green lines) and the associated energy difference with the full electronic energy (golden lines).

The final piece of evidence to unveil the reasons behind the conformational preferences of methyl groups in caffeine is also afforded by NBO in that the sum of all $E_{d\to a}^{\left(2\right)}$ for the M1 conformation which places all C–H bonds out of plane ($\phi_{1}$ = 30${}^{\circ }$, Figure 6) is 2.5 kcal/mol lower than for **I**, which evidently supports the maximization of charge transfer interaction energies for the preferred conformation. In summary, NBO provides solid evidence to explain conformational preferences for the methyl groups in caffeine because when C–H bonds from the methyl groups eclipse C=O bonds (M1, M2) or C–H bonds (M3), the magnitudes of the donor → acceptor interaction energies are maximized in both directions.

Under a purely quantitative perspective, the preferred conformation of M3, eclipsing a methyl C–H bond with a ring C–H, affords the largest donor→acceptor hyperconjugation energies (Figure 4). Thus, although energy barriers for all methyl rotations are quite small (0.25, 0.15, 0.43 kcal/mol for M1, M2, M3, respectively [62] and similar values obtained here as listed in Table 1), M3 stands as the most important group in determining the overall structure of caffeine.

We also deleted all NL orbitals for the **I→II→I** and **I→III→I** interconversions and show the resulting relaxed scans in Figure 6, which contains the following plots: the full electronic energy relaxed scans (blue curves), the scans for the Lewis structures (green curves) and a quantification of the hyperconjugation effect as the difference between the energies of the pure Lewis structure and the full electronic energies (golden curves). The minima at both green and golden profiles in Figure 6 ($\phi_{1},\phi_{2}$ = 30${}^{\circ }$, 90${}^{\circ }$) correspond to structures where all three C–H bonds in M1 or M2 are located off-plane, as the classical steric repulsion would indicate. However, since these structures are not stationary points within the blue profiles, this is clear evidence that hyperconjugation overcomes steric hindrance and leads to C–H eclipsing with C=O in the preferred conformation of M1, and to C–H eclipsing with C=O or with the aromatic ring in the preferred conformations of M2. This observation is yet another example in a long list of catastrophic failures that plague general and “advanced” chemistry books when rationalizing chemical bonding and structural preferences, failures that are a consequence of too primitive and inadequate classical treatments. Weinhold himself has championed a revision of these issues, [30,31,32] specifically asking “*when will chemistry textbooks begin to serve as aids, rather than barriers, to this enriched quantum–mechanical perspective on how molecular turnstiles work?*” [30]. 

### 3.5. Hyperconjugation Effects on Molecular Geometries

**Figure 7:**
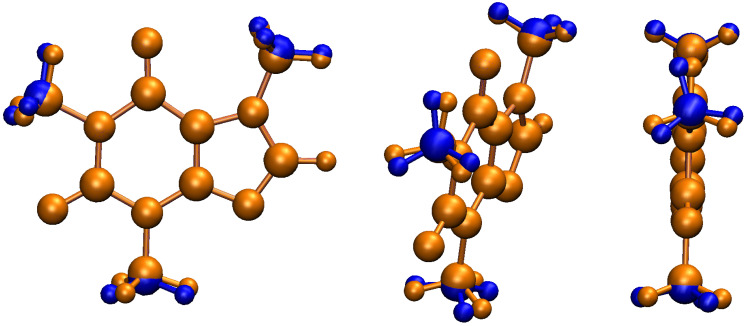
Hyperconjugative effects on the molecular geometry of caffeine. Selectively removing all non-Lewis orbitals involved in the bidirectional –CH${}_{3}⇆$ aromatic charge transfer leads to an enlargement of the C–N bonds in the H${}_{3}$C–N groups and to out of plane C–H bonds (blue structures).

NBO7 imposes a limit of 50 geometrical variables that can be optimized upon deletion of hyperconjugative interactions, thus, to stay under this limit, in this work we selectively removed all non–Lewis orbitals involved in the bidirectional –CH${}_{3}⇆$ aromatic charge transfer. Notice that under these circumstances, only the four bonds in each H${}_{3}$C–N group, as well as the associated angles and dihedrals, are allowed to change. These selective deletions lead to geometrical changes as shown in Figure 7. The observed geometrical changes uncover the delicate interplay between hyperconjugation and more classical concepts such as steric repulsion and electrostatic interactions in molecular structures. Two significant effects are observed. First, there is a sensible enlargement of up to 8% in the C–N bond distances in the resulting structures. Second, no C–H bond lies now in the heavy atom plane, with deviations from planarity of ≈18${}^{\circ }$, 28${}^{\circ }$, 3${}^{\circ }$ for M1, M2, and M3, respectively, as measured by the corresponding H–C–N–C dihedral. These results add solid support to the insight gathered from the above analyzed scans (Figure 6) and conclusively show that in the absence of the –CH${}_{3}⇆$ aromatic bidirectional charge, steric repulsion would dictate the conformation of the methyl groups, in other words, the seemingly modest orbital interactions between the methyl groups and the aromatic system, overcome the a priori stronger steric repulsion to finally determine the structure of caffeine.

### 3.6. QTAIM and NCI Analysis

Our results indicate that for the particular case of the methyl rotation in caffeine, both QTAIM and NCI afford misleading results which actually strengthen the NBO picture and lead us to state that conformational preferences in caffeine are exclusively due orbital interactions. This conclusion is based on the following observations:

1. Within the respective thresholds, both QTAIM and NCI fail to detect any intramolecular interactions associated to the stabilization of the eclipsed C–H/C–H conformations (Structures **I–IV**, Figure 2) while NBO clearly identifies bidirectional –CH${}_{3}\;⇆$ aromatic charge transfer as the mechanism behind this conformational preference (bottom row of Figure 5).
2. Bonding paths are obtained only for the M3⋯Carbonyl interactions (Structures **V-VII**, Figure 2). Thus, QTAIM suggests a wrong conformation for M3 favoring the 1,4-allylic effect over the C-H/C-H eclipsing and even over the 1,3-allylic effect while saying nothing about the conformational preferences of M1 and M2. The very small accumulation of electron density at the bond critical points, $\rho \left(r_{c}\right)\approx 1.2\times 10^{-2}$ a. u., about half that of the water dimer [43,44], a well studied weakly bonded system, is a good descriptor of the tiny rotational barriers.
3. NCI affords green (with a small amount of red) surfaces for all conformations of M1 and M2, and only for the M3 conformation which eclipses a carbonyl group (Figure 2). However, the sizes of the surfaces suggest that the wrong conformation of M3 should be preferred since nothing can be inferred from structure **I–IV**.

### 3.7. Methyl Rotation and Biological Activity

**Figure 8:**
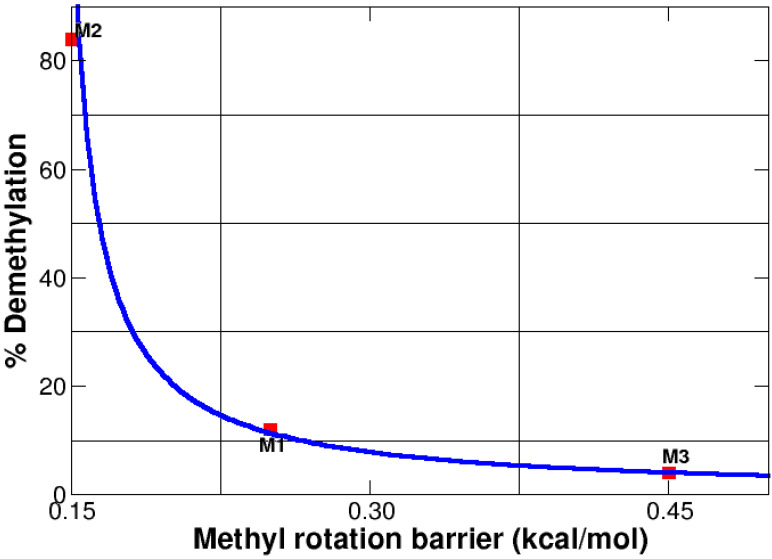
Ability to eliminate methyl groups in caffeine as a function of the rotational barrier. Experimental data (red squares) taken from Ref. [87].

Caffeine is metabolized in the liver via single demethylation by the cytochrome P450 oxidase enzyme [84,85,86]. Gratifyingly, despite being an enzymatic reaction, there is an inverse correlation (see Figure 8) between the size of the internal rotational barriers for the methyl groups and their ability to demethylate, thus, smaller barriers are tied to better leaving groups. It is well established that each demethylation produces a different active derivative, therefore, demethylation of M2 (the smallest barrier) yields paraxantine, which increases lipolysis and leads to high levels of fatty acids in blood, demethylation of M1 (the intermediate barrier) yields theobromine, which dilates blood vessels, and elimination of M3 (the largest barrier) yields theophylline, which is a muscle relaxant. The reader is advised to recognize that correlation does not mean causation, nonetheless, all the evidence provided in this manuscript, leads us to establish a strong link between hyperconjugative effects affecting methyl rotations and the biological activity of caffeine and its demethylated derivatives.

## 4. Summary and Conclusions

The most important conclusion of this work, which removes all experimental ambiguities in the assignation of the molecular geometry, is that bidirectional –CH${}_{3}\;⇆$ aromatic charge transfer is responsible for the three methyl groups in caffeine adopting uncommon conformations, each one placing a C–H bond on the same plane of the aromatic ring.

Aided by tiny rotational barriers, no larger than 1.3 kcal/mol at the DLPNO–CCSD(T) level, which render each methyl group a free rotor or a fluxional conformation at room conditions, the equilibrium conformations overcome steric repulsion to yield 1,3 C–H/C=O, C–H/C–N, and C–H/C–H bond eclipsation. The most significant contributors to the hyperconjugative effect driving structural preferences in caffeine within the NBO donor → acceptor orbital paradigm are $n_{N}\to \sigma_{C-H}^{*}$, $n_{O}\to \sigma_{C-H}^{*}$, $\sigma_{N-C}\to \sigma_{C-H}^{*}$, $\sigma_{C-H}\to \sigma_{N-C}^{*}$. For each methyl group, the geometrical arrangement placing one C–H bond in the aromatic plane maximizes the magnitudes of the donor → acceptor interactions. Inclusion of solvent effects via continuum models make no difference in the conformational preferences or in the energetics of the problem. For the particular case of methyl rotation in caffeine, QTAIM and NCI afford negative results, effectively providing strong support to the observation that orbital interactions are exclusively behind the conformational preferences that place C–H bonds in the aromatic plane, leading to the anomalous eclipsing of C–H bonds with large groups.

## Figures and Tables

**Table 1:** Gas phase and solution energy differences in kcal/mol with respect to **I**, the lowest energy structure, for all caffeine conformers shown in Figure 2. ΔE: purely electronic energies. ΔE${}_{\mathrm{ZPE}}$: electronic energies corrected by the B3LYP–D3 ZPE. ΔΔG: relative Gibbs energies at room conditions. Basis set: 6–311++G(d,p). $n_{i}$: number of imaginary frequencies. CCSD(T) energies calculated under the DLPNO–CCSD(T) formalism [68,69].

Conformer	Dihedral		$n_{i}$	CCSD(T)		B3LYP–D3			Water		Acetone		Acetonitrile		Chloroform		Dichloromethane		Toluene	
	$\phi_{1}$	$\phi_{2}$		ΔE	ΔE${}_{\mathrm{ZPE}}$	ΔE	ΔE${}_{\mathrm{ZPE}}$	ΔΔG	ΔE${}_{\mathrm{ZPE}}$	ΔΔG	ΔE${}_{\mathrm{ZPE}}$	ΔΔG	ΔE${}_{\mathrm{ZPE}}$	ΔΔG	ΔE${}_{\mathrm{ZPE}}$	ΔΔG	ΔE${}_{\mathrm{ZPE}}$	ΔΔG	ΔE${}_{\mathrm{ZPE}}$	ΔΔG
**I**	0	180	0	0.0	0.0	0.0	0.0	0.0	0.0	0.0	0.0	0.0	0.0	0.0	0.0	0.0	0.0	0.0	0.0	0.0
**II**	60	180	1	0.3	0.2	0.3	0.2	1.1	0.2	1.0	0.2	0.9	0.2	1.0	0.2	0.9	0.2	0.9	0.2	0.9
**III**	0	120	1	0.4	0.3	0.2	0.1	0.8	0.1	1.0	0.1	0.9	0.1	1.0	0.1	0.9	0.1	0.9	0.1	1.0
**IV**	60	120	2	0.8	0.6	0.5	0.3	2.2	0.3	1.9	0.3	1.9	0.3	1.9	0.3	1.9	0.3	1.8	0.3	0.3
**V**	0	180	1	0.8	0.8	0.4	0.4	2.1	0.6	1.2	0.6	1.2	0.6	1.2	0.6	1.4	0.6	1.3	0.6	1.6
**VI**	60	180	1	1.1	1.0	0.7	0.6	1.8	0.8	2.3	0.8	2.3	0.8	2.3	0.8	1.1	0.8	0.8	0.7	1.4
**VII**	0	0	2	1.1	1.1	0.6	0.5	3.1	0.8	2.3	0.7	2.3	0.8	2.3	0.7	2.3	0.7	2.3	0.7	2.6
**VIII**	180	0	2	1.5	1.3	0.9	0.7	2.6	0.9	3.2	0.9	3.2	0.9	3.2	0.9	2.1	0.9	1.9	0.9	2.3

**Table 2:** Energy of NBO deletions in kcal/mol for all conformers of caffeine in Figure 2. Indiscriminate deletion: $\Delta E\left(L\right)=E{\left(L\right)}_{conformer}-E{\left(L\right)}_{I}$: considering the fully localized Lewis structure (erasing all non–Lewis orbitals from the Fock matrix). ΔE(NL) = ${\left(E_{full}-E\left(L\right)\right)}_{conformer}-{\left(E_{full}-E\left(L\right)\right)}_{I}$. Selective deletion: deleting all interactions when the methyl group acts as donor or as acceptor of charge.

Conformer	ΔE(L)	ΔE(NL)	M1		M2		M3	
			Donor	Acceptor	Donor	Acceptor	Donor	Acceptor
**I**	0.0	0.0	6.8	10.6	7.5	9.5	8.8	8.8
**II**	−0.4	0.7	6.7	10.5	7.4	9.5	8.8	8.8
**III**	0.6	−0.4	6.8	10.6	7.4	9.5	8.8	8.8
**IV**	0.1	0.5	6.7	10.5	7.3	9.5	8.8	8.8
**V**	−0.8	1.2	6.8	10.6	7.5	9.5	8.6	9.6
**VI**	−1.2	1.8	6.7	10.5	7.4	9.5	8.6	9.6
**VII**	−0.2	0.8	6.8	10.6	7.4	9.5	8.6	9.6
**VIII**	−0.6	1.5	6.7	10.5	7.3	9.5	8.6	9.6

## Data Availability

Cartesian coordinates for the optimized conformers in Figure 2 are provided in the Supplementary Material.

## Supplementary Materials

The following supporting information can be downloaded at: https://www.mdpi.com/article/10.3390/molecules27061937/s1, Cartesian coordinates for the optimized conformers in Figure 2.

## Abbreviations

The following abbreviations are used in this manuscript:

NBO	Natural Bond Orbitals
NL	Non–Lewis Orbitals
DFT	Density Functional Theory
DLPNO–CCSD(T)	domain-based local pair natural orbital coupled-cluster
QTAIM	Quantum Theory of Atoms in Molecules
NCI	Non–Covalent Interactions
