# Supplementary material for: Analysis of Conformational Preferences in Caffeine
Source: Molecules. 2022 Mar 17;27(6):1937. doi: 10.3390/molecules27061937 (PMC8949453; doi:10.3390/molecules27061937)
Supplement: Supplementary file 1 [file molecules-27-01937-s001.zip › molecules-1463732-supplementary.pdf]

```
24
I Energy=   -680.584790529      Hartrees
  N        1.247565   -1.106689    0.000075
  C        1.853775    0.163739    0.000026
  N        1.013563    1.270389    0.000100
  C       -0.349039    1.091399    0.000058
  C       -0.907080   -0.171084    0.000086
  C       -0.142374   -1.379883    0.000009
  N       -2.277591    0.037107   -0.000090
  C       -2.445750    1.381119    0.000012
  N       -1.301758    2.058401   -0.000058
  C        2.180881   -2.240572    0.000007
  O        3.066355    0.286225   -0.000027
  C        1.624833    2.601105   -0.000058
  O       -0.592781   -2.518368   -0.000133
  C       -3.311394   -0.992318    0.000053
  H       -3.426840    1.831795   -0.000023
  H        1.592575   -3.153489   -0.000096
  H        2.816830   -2.196004    0.884308
  H        2.816907   -2.195838   -0.884227
  H        0.824527    3.336899    0.000151
  H        2.249907    2.722160   -0.885334
  H        2.250361    2.722134    0.884896
  H       -4.285276   -0.503359   -0.001073
  H       -3.212240   -1.620563    0.884800
  H       -3.210908   -1.622070   -0.883455
24
II Energy=   -680.584339663      Hartrees
  N        1.250785    1.105003   -0.000778
  C        1.864608   -0.165026    0.000171
  N        1.018843   -1.268508   -0.000373
  C       -0.344690   -1.093116   -0.000512
  C       -0.906596    0.166995   -0.000374
  C       -0.137142    1.371880   -0.000166
  N       -2.276072   -0.044058   -0.000342
  C       -2.440864   -1.388853   -0.000452
  N       -1.295049   -2.062891   -0.000612
  C        2.124579    2.286236   -0.000243
  O        3.075339   -0.305385    0.001221
  C        1.628363   -2.600552    0.000244
  O       -0.569443    2.517410    0.000651
  C       -3.311153    0.983924    0.000705
  H       -3.420780   -1.842050   -0.000513
  H        1.929626    2.884841    0.890141
  H        1.911754    2.897376   -0.877717
  H        3.155327    1.947477   -0.012400
  H        0.826817   -3.334941   -0.000318
  H        2.252834   -2.722287    0.885764
  H        2.254201   -2.722420   -0.884285
  H       -4.284582    0.494121   -0.003505
  H       -3.209929    1.616023   -0.881107
  H       -3.214608    1.609902    0.887458
24
III Energy=   -680.584509225      Hartrees
  N        1.263651   -1.092094   -0.000146
  C        1.861058    0.184258   -0.000115
  N        1.003373    1.281546   -0.000466
  C       -0.354989    1.080203   -0.000191
  C       -0.902617   -0.185650   -0.000168
  C       -0.122882   -1.384306    0.000100
  N       -2.273904    0.011964    0.000193
  C       -2.452494    1.355346    0.000251
  N       -1.313382    2.041564    0.000087
  C        2.208725   -2.216865    0.000007
  O        3.073726    0.302745    0.000110
  C        1.542863    2.642658    0.000064
  O       -0.558131   -2.528443    0.000328
  C       -3.299547   -1.025627   -0.000196
  H       -3.437318    1.797819    0.000461
```



```
  H        1.629591   -3.135554    0.000142
  H        2.844212   -2.165686    0.884186
  H        2.844184   -2.165932   -0.884210
  H        1.201657    3.174512    0.889547
  H        1.194184    3.177694   -0.884534
  H        2.626668    2.573815   -0.004436
  H       -4.277345   -0.544615    0.003059
  H       -3.192775   -1.655834    0.882259
  H       -3.196687   -1.651590   -0.886178
24
IV Energy=   -680.583959073      Hartrees
  N       -1.268056   -1.089346   -0.000854
  C       -1.872708    0.187287   -0.000024
  N       -1.007899    1.280589   -0.000019
  C        0.351230    1.081408   -0.000365
  C        0.902040   -0.182454   -0.000537
  C        0.116836   -1.376989    0.000096
  N        2.272514    0.017341    0.000013
  C        2.448597    1.361587    0.000260
  N        1.307951    2.045017    0.000080
  C       -2.154112   -2.262249   -0.000531
  O       -3.083119    0.324514    0.000544
  C       -1.542727    2.644203    0.000210
  O        0.532085   -2.528246    0.001265
  C        3.298932   -1.019255   -0.000402
  H        3.432662    1.805949    0.000475
  H       -1.965151   -2.862694    0.890008
  H       -1.946738   -2.875573   -0.877889
  H       -3.181617   -1.914250   -0.013109
  H       -1.200126    3.174572   -0.889701
  H       -1.190301    3.177609    0.884374
  H       -2.626703    2.580214    0.006088
  H        4.276577   -0.537766    0.002339
  H        3.196413   -1.645476   -0.886436
  H        3.193149   -1.649157    0.882594
24
V Energy=   -680.584106516      Hartrees
  N       -1.254699   -1.101433   -0.000223
  C       -1.855659    0.171807   -0.000283
  N       -1.008652    1.272106   -0.000179
  C        0.352987    1.084759   -0.000134
  C        0.908792   -0.179418   -0.000224
  C        0.132946   -1.383582   -0.000225
  N        2.281486    0.028982    0.000046
  C        2.449948    1.372844    0.000052
  N        1.306844    2.050625    0.000098
  C       -2.194710   -2.230160    0.000088
  O       -3.067450    0.300058    0.000342
  C       -1.611563    2.606745    0.000178
  O        0.568889   -2.528566    0.000138
  C        3.330707   -0.991721   -0.000019
  H        3.430954    1.824355    0.000164
  H       -1.612543   -3.146938   -0.000318
  H       -2.830837   -2.181265   -0.883922
  H       -2.829994   -2.181573    0.884731
  H       -0.806966    3.337830   -0.000022
  H       -2.235832    2.731362    0.885620
  H       -2.236437    2.731504   -0.884812
  H        2.853075   -1.968575    0.000306
  H        3.950996   -0.888926    0.891943
  H        3.950538   -0.889302   -0.892344
24
VI Energy=   -680.583720599      Hartrees
  N       -1.256739    1.100577    0.000203
  C       -1.866267   -0.172316    0.000224
  N       -1.014849   -1.269994    0.000162
  C        0.348157   -1.087413    0.000097
  C        0.908044    0.174018    0.000149
  C        0.128931    1.375091    0.000093
```

```
  N        2.279470   -0.037132   -0.000076
  C        2.444497   -1.381807   -0.000105
  N        1.299342   -2.056260   -0.000110
  C       -2.136760    2.277446   -0.000002
  O       -3.076203   -0.317097   -0.000193
  C       -1.617260   -2.605376   -0.000112
  O        0.549156    2.526042   -0.000140
  C        3.328557    0.983460    0.000020
  H        3.424168   -1.836136   -0.000210
  H       -3.165757    1.932865    0.000427
  H       -1.936410    2.883139   -0.884319
  H       -1.935852    2.883787    0.883736
  H       -0.812166   -3.335903   -0.000094
  H       -2.241711   -2.730325   -0.885324
  H       -2.241957   -2.730539    0.884892
  H        2.850331    1.960196   -0.000267
  H        3.949104    0.881046   -0.891829
  H        3.948665    0.881355    0.892212
24
VII Energy=   -680.583854050      Hartrees
  N        1.270508   -1.086887   -0.000151
  C        1.862791    0.192105   -0.000619
  N        0.998585    1.283061   -0.000236
  C       -0.358713    1.073766   -0.000155
  C       -0.904245   -0.193653   -0.000095
  C       -0.113740   -1.387678   -0.000170
  N       -2.277570    0.004114   -0.000000
  C       -2.456525    1.347344   -0.000132
  N       -1.318224    2.034098   -0.000017
  C        2.222106   -2.206594    0.000295
  O        3.074631    0.316435    0.000354
  C        1.529681    2.647484    0.000010
  O       -0.534793   -2.538004    0.000111
  C       -3.318191   -1.025322    0.000313
  H       -3.441267    1.790644   -0.000095
  H        1.649026   -3.129044    0.000532
  H        2.857153   -2.151260    0.884653
  H        2.857302   -2.151782   -0.883987
  H        2.614041    2.585038   -0.000172
  H        1.181738    3.178736    0.887276
  H        1.181474    3.179119   -0.886920
  H       -2.832208   -1.998151   -0.000108
  H       -3.939430   -0.927890   -0.891517
  H       -3.938592   -0.928258    0.892769
24
VIII Energy=   -680.583366377      Hartrees
  N       -1.273873   -1.084716    0.000008
  C       -1.873782    0.194193    0.000358
  N       -1.003927    1.281850    0.000128
  C        0.354504    1.075959    0.000059
  C        0.903390   -0.189142   -0.000023
  C        0.108418   -1.379074    0.000034
  N        2.275619    0.010542   -0.000006
  C        2.452041    1.354499    0.000099
  N        1.312072    2.038599    0.000045
  C       -2.165442   -2.253709   -0.000241
  O       -3.083727    0.335991   -0.000047
  C       -1.531474    2.648317    0.000111
  O        0.511870   -2.535780   -0.000043
  C        3.315403   -1.019499   -0.000254
  H        3.435765    1.799999    0.000109
  H       -3.191206   -1.900397   -0.000647
  H       -1.970520   -2.861434    0.883887
  H       -1.969814   -2.861555   -0.884123
  H       -2.615796    2.589932    0.000288
  H       -1.181255    3.178358   -0.886961
  H       -1.180975    3.178467    0.887007
  H        2.828223   -1.991911    0.000129
  H        3.936792   -0.922984    0.891593
```



```
  H        3.936067   -0.923335   -0.892647
```
